# Treatment of Progressive Cherubism during the Second Dental Transitional Phase with Calcitonin

**DOI:** 10.1155/2023/2347855

**Published:** 2023-11-09

**Authors:** Ethan Meijer, Henk van den Berg, Arjen H. G. Cleven, Esther Edelenbos, Willem H. Schreuder

**Affiliations:** ^1^Department of Oral and Maxillofacial Surgery, Amsterdam UMC (Location AMC) and Academic Center for Dentistry Amsterdam, University of Amsterdam, Amsterdam, Netherlands; ^2^Department of Pediatric Oncology, Amsterdam University Medical Centers Location AMC, University of Amsterdam, Amsterdam, Netherlands; ^3^Department of Pathology, University Medical Center Groningen, University of Groningen, Groningen, Netherlands

## Abstract

Cherubism is an autosomal dominant disease with variable expression. Aggressive forms of untreated cherubism may lead to severe malformation of the maxillofacial skeleton, developing tooth germs and teeth. Scarcely described and empirically applied interventional therapies during active stages of the disease try to limit the damage and deformation caused by progression of expanding intraosseous lesions. The final goal is to minimize the need for corrective surgeries once progressive growth has halted and disease enters its quiescent phase. New insights into the pathophysiology of cherubism hypothesize a potential role for dental development and jaw growth in the (hyper)activation of the disease. Theoretically, this could guide the ideal moment of pharmacological interventions. In this case report, the off-label use of systemic calcitonin treatment is described, stressing particularly the potential importance of its appropriate timing and duration of treatment.

## 1. Introduction

Cherubism (OMIM 118400) is an autosomal dominant disease with a highly variable expression [1–3]. Patients show painless bilateral expansion of the mandible and/or maxilla, manifesting itself in early childhood. Subsequently, lesions show progressive growth into puberty, whereafter partial or full regression occurs. Radiological imaging shows expansive, bilateral multilocular well-defined radiolucent lesions, as well as displaced or missing teeth or/and tooth germs [1–3]. Aggressive forms of untreated cherubism lead to undesirable damage, potentially resulting in a lifelong impact on quality of life.

The disease is associated with a genetic mutation of *SH3BP2* (SRC Homology 3 Domain Binding Protein 2) located on chromosome 4p16 [4, 5]. In mouse models, this mutation leads to a gain of function of SH3BP2 causing an autoinflammatory osteolytic bone disorder as a result of hyperactive osteoclasts and hyperreactive macrophages producing large amounts of TNF-alpha [5].

Despite identification of the basic genetic background, the treatment for cherubism is still under debate. Several experimental efforts have been made using calcitonin administrations [3]. These efforts are based on calcitonin therapy as applied with varying degrees of success in the treatment of other giant cell-rich lesions of the jaw [6].

Novel research investigated the relationship between cherubism and its age-dependent symptomatology including the role of tooth development and jawbone remodeling [7]. This led to new insights suggesting that exposure to endogenous and exogenous molecules encountered by the innate immune system during jaw growth and dental eruption is an important factor for (hyper)activation of the disease [7, 8].

Therefore, we hypothesized that calcitonin treatment given during one or even both phases of the dental transitional periods might prevent the most severe progression of cherubism and limit its associated morbidity. In this case report, we demonstrate a patient with severe cherubism who was treated with systemic calcitonin during the second transitional phase in order to maintain normal tooth development and facial growth.

## 2. Case Report

An 11-year-old boy was referred to assess progression of previously diagnosed cherubism. DNA analysis revealed a heterozygous germline mutation of c.1253C>A (p.Pro418His) in exon 9 of chromosome 4p16. After diagnosis at the age of 6, active surveillance showed minimal progression of the swelling. However, since the start of the second transitional phase of his dental development, more rapid progression was observed.

**Figure 1:**
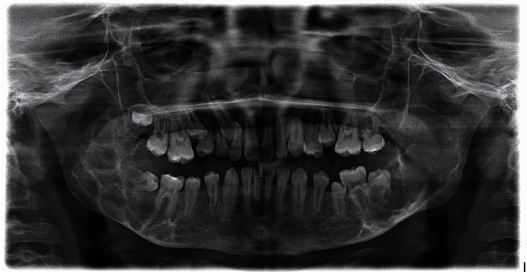
Orthopantomography demonstrating bilateral poorly defined multilocular radiolucent areas in the mandible involving the posterior body, angle, and coronoid process. There was impaction of several teeth in the maxilla and the second molars in the lower jaw. There was root resorption of the tooth #18, 19, and 31.

**Figure 2:**
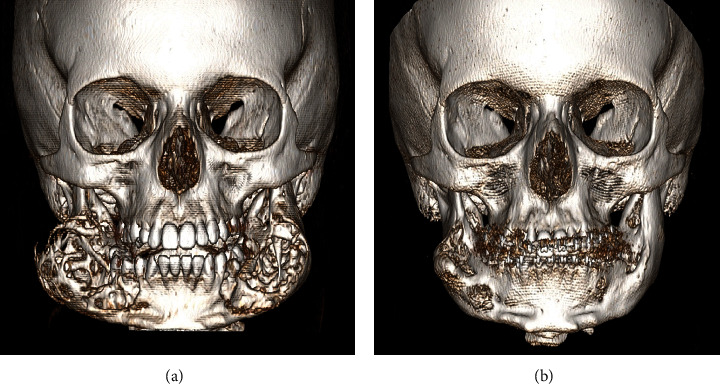
3D reconstruction of computed tomography at start of treatment (a) and after 35 months of treatment (b). Notice the massive lateral expansion of the lesions as well as severe cortical thinning at start of treatment. After 35 months, lesions were reduced in size and largely ossified facilitating future corrective surgery.

Apart from cherubism, he was in good general health. At the time of presentation, clinical examination demonstrated painless, symmetrically swollen cheeks. No exophthalmos or scleral show below the iris was observed. Dental development was in the mixed dentition phase, and the palate was slightly high arched. Orthopantomography (OPT) showed characteristic features of cherubism (Figure 1), which were confirmed by computed tomography (CT) (Figure 2(a)). Based on these findings, a cherubism Motamedi-Raposo grade III Class 5 [9] was diagnosed.

Predominantly for psychosocial considerations, surgical correction of facial disfigurement was requested. Because of the risks of severe intraoperative hemorrhage, severe thinning of the cortex surrounding the lesions, and recurrent progression after surgical intervention, systemic therapy with subcutaneous injections of calcitonin (Calcitonine EssPharma, Essential Pharma Ltd., Birkirkara, Malta) 100 IU/day was initiated. CT scans were repeated every 6 months to evaluate the effect. The treatment was well tolerated without side effects, apart from local injection site pain and nausea in the first week of treatment.

After 6 months, clinical progression of the lesions was halted. A CT scan showed decrease in the size of the lesions, mild intralesional calcifications, and repair of the cortex resembling encapsulation. After 12 months, clinical regression of the palpable lesions was noted. A CT scan demonstrated a volume decrease from 20 ml to 7 ml on the left side and from 25 ml to 20 ml on the right.

Because of the positive response and good tolerance of calcitonin, therapy was extended up to 35 months. Clinically, the swelling on both sides of the face continued to decrease. Control CT (Figure 2(b)) demonstrated a significant reduction in size and ossification of the lesions, resulting in less prominent facial features characteristic for cherubism while normal dental development continued. Considering the results obtained, it was agreed that contour corrections were to be postponed after completion of skeletal growth.

## 3. Discussion

Based on a combination of clinical behavior and radiographic findings, cherubism is classified into quiescent, nonaggressive, and aggressive [10]. Quiescent cherubic lesions occur in older patients and should be considered remnants of earlier disease; nonaggressive lesions are minimally progressive and present in older teenagers once dental development and eruption is almost completed. However, the aggressive form is characterized by large, rapidly growing lesions accompanied by tooth displacement, root resorption, and cortical bone thinning. These lesions occur in young children before and during puberty. They present a significant challenge, as treatment may be warranted to prevent excessive facial dysmorphology and long-term sequelae.

Surgical intervention is preferably postponed until lesions enter the quiescent phase because of the high risk on regrowth [3]. In the aggressive phase, pharmacological treatment could be an alternative treatment option to stabilize the disease and delay or prevent surgical intervention. A variety of empirical pharmacological therapies has been described with various levels of success, including bisphosphonates, calcitonin, corticosteroids, denosumab, imatinib, interferons, TNF inhibitors, and tacrolimus [3, 11]. Existing case reports on calcitonin administration describe positive outcomes when treatment duration exceeded one year [3]. Failed therapies were associated with either a shorter duration of treatment or the occurrence of side effects.

New insights into the pathophysiology of cherubism, generating hypotheses on the age-related autoinflammation and macrophage activation, underline the potential importance of timing systemic treatment to achieve a successful outcome. Yoshitaka et al. [7] suggest that aggressive cherubism is a result of hyperactivation of the TLR2/TLR4 and MYD88 (myeloid factor-88) pathway through increased DAMP (damage-associated molecular pattern) and PAMP (pathogen-associated molecular pattern) production, leading to overproduction of TNF-alpha by hyperactive macrophages. DAMPs are released by stressed or dead cells; this might be increased during the phase of active tooth eruption. PAMPs could be increased due to the change in oral microbioma during childhood. This hypothesis explains the onset of mandibular enlargement in our patient during his first transitional period around the age of six years, the minimal progression thereafter, and the rapid progression during his second transitional phase at the age of eleven years.

Our patient adds to the earlier reported positive effect of calcitonin treatment on cherubism and demonstrates that pharmacological therapy can be effective. As duration of treatment is mentioned to be influential on outcome, we stress that close guidance of treatment by a dedicated team is important to manage potential side effects and achieve good compliance. The results achieved in the current case potentially also underline the importance of appropriate timing of systemic treatment and suggest that treatment guided by the transitional phases could increase successful outcomes.

It should be noted that extended calcitonin treatment was associated with a small increased cancer risk in adults, whereupon the European Medicines Agency (EMA) advised against long-term treatment and decided to narrow down therapy indications [12]. While the association was proven, its causality was not as clear, but was suggested to be the result of increased tumour growth progression rather than oncogenesis. This could possibly mean that the increased risk the EMA decision is based on, might not be applicable to children to the same extent as adult patients. Yet, the long-term effects of calcitonin remain unknown in paediatrics. For the off-label use of calcitonin in cherubism, we therefore recommend carefully assessing the risk-benefit ratio for every individual treatment.

## Data Availability

The corresponding author can be approached to request additional data.
